# Preclinical antitumor activity of ST7612AA1: a new oral thiol-based histone deacetylase (HDAC) inhibitor

**DOI:** 10.18632/oncotarget.3240

**Published:** 2014-12-25

**Authors:** Loredana Vesci, Elena Bernasconi, Ferdinando Maria Milazzo, Rita De Santis, Eugenio Gaudio, Ivo Kwee, Andrea Rinaldi, Silvia Pace, Valeria Carollo, Giuseppe Giannini, Francesco Bertoni

**Affiliations:** ^1^ Research & Development, Sigma-Tau, Pomezia, Italy; ^2^ Lymphoma and Genomics Research Program, IOR Institute of Oncology Research, Bellinzona, Switzerland; ^3^ Dalle Molle Institute for Artificial Intelligence (IDSIA), Manno, Switzerland; ^4^ Hysto-Cyto Service srl, Rome, Italy; ^5^ Lymphoma Unit, IOSI Oncology Institute of Southern Switzerland, Bellinzona, Switzerland

**Keywords:** histone deacetylase inhibitor, anti-tumor, oral, preclinical, tumor models

## Abstract

ST7612AA1 (property of Sigma-Tau), a thioacetate-&omega; (&gamma;-lactam amide) derivative, is a potent, second generation, oral pan-histone deacetylase inhibitor (HDACi). Aim of the study was to assess the efficacy of ST7612AA1 in solid and haematological tumors, and to characterize its mechanism of action. *In vitro*, ST7612AA1 potently inhibited different class I and class II HDACs, leading to restore the balance of both histone and non-histone protein acetylation. *In vivo*, it induced significant anti-tumor effects in xenograft models of lung, colon, breast and ovarian carcinomas, leukemia and lymphoma. This was likely due to the modulation of different HDAC substrates and induction of transcriptional changes with respect to several genes involved in key processes, such as cell cycle regulation, DNA damage checkpoints, immune response, cell adhesion and epithelial-to-mesenchymal transition. PK analysis confirmed the pro-drug nature of ST7612AA1, which is rapidly absorbed and converted to ST7464AA1 after a single oral dose in mice. ST7612AA1 was selected from a novel generation of oral HDAC inhibitors. Its high efficacy correlated with its potent and selective inhibitory activity of HDAC and was combined with a favorable pharmacodynamics profile. These aspects support a clinical development of ST7612AA1 towards a broad spectrum of human solid and haematologic malignancies.

## INTRODUCTION

Epigenetic mechanisms result in changes in gene expression without altering the DNA sequence per se. These changes involve DNA methylation and histone modifications (such as acetylation), which are potentially reversible. Due to this property, modulation of epigenetic gene suppression has become a very attractive model to treat cancer [1]. The expression of histone deacetylases (HDAC) is frequently altered in several malignancies [2], thus histone deacetylase inhibitors (HDACis) able to bind with high affinity to HDACs and to severely affect their enzymatic activity, have emerged as a promising new class of multifunctional anticancer drugs [3, 4]. In fact, HDACis have been previously shown to reduce multiple epigenetic pathways exerting pro-tumorigenic activity. In addition to regulate gene expression and transcription through chromatin remodelling, HDACis can also modulate a variety of cellular functions including growth, differentiation, and survival [5, 6], by enhancing acetylation of a wide variety of proteins, including transcription factors, modular chaperones, and structural components [3, 7]. Specifically, HDACis have been shown to induce several down-stream effects in tumor cell lines, including: cell cycle arrest, induction of apoptosis, inhibition of angiogenesis, activation or inactivation of tumor suppressor genes or oncogenes, and decrease of invasion and metastasis [3, 4, 8]. Interestingly, tumor cells appear much more sensitive to the induction of apoptosis by HDAC inhibitors than normal cells, probably linked to the disturbed chromatin structure in cancer cells [9] and to the induction of double-strand DNA breaks [10]. The classical HDAC inhibitors inhibit the function of one or more of the 11 known zinc-containing HDAC enzymes. The zinc-containing HDAC enzymes can be classified into several Classes: Class I HDAC (HDAC1, 2, 3, 8), Class IIA (HDAC4, 5, 7, 9), Class IIB (HDAC6, 10) and Class IV (HDAC11) [11]. Class III HDACs or Sirtuins, have a different catalytic mechanism and are not a target for the classical HDAC inhibitors. Generally, pan-HDAC inhibitors inhibit HDACs from Class I, II and IV, while Class specific-HDAC inhibitors only inhibit HDACs from either Class I or Class II. At the present, three HDACis – vorinostat (suberoylanilide hydroxamic acid, Zolinza) orally delivered, depsipeptide (romidepsin, Istodax) and belinostat (Beleodaq) intravenously delivered– have received approval from the US Food and Drug Administration (FDA) for treatment of refractory cutaneous T-cell lymphoma (CTCL), and more recently, depsipeptide has gained FDA approval for peripheral T-cell lymphoma (PTCL) [12-14]. Several HDAC inhibitors are under clinical development in various malignancies, many of them of haematological origin, such as leukemia, lymphoma, and myelodysplastic syndrome [2, 15]. Broadly, HDACis can be classified into different structural groups: the hydroxamic acids, cyclic peptides, benzimides and short-chain fatty acids. Although HDAC inhibitors preferentially targeting a single HDAC have been recently developed [16], it is noteworthy that the hydroxamates are able to target and affect all classes of HDACs, thus exerting nonspecific HDAC-inhibition activity [17, 18].

**Figure 1:**
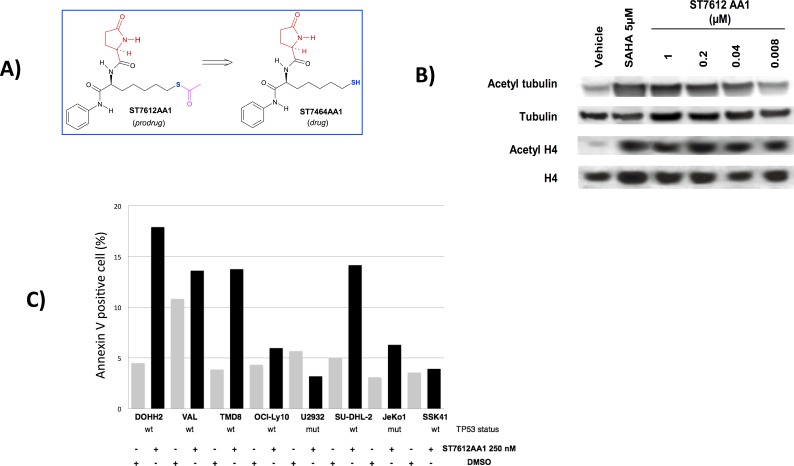
ST7612AA1 reduces HDAC activity and induces apoptosis of human cancer cells A) Chemical structure of the prodrug ST7612AA1 and its drug ST7464AA1. B) Assessment of a dose-dependent effect of ST7612AA1 on acetylation of alpha-tubulin and histone H4 in NCI-H460 NSCLC cells after 3 h exposure. SAHA 5 μM was used as internal positive reference. To control for equal loading, blots were stripped and reprobed with antibodies against tubulin and histone H4. C) Assessment of ST7612AA1-induced apoptosis in lymphoma cell lines. Y-axis, percentage of Annexin V positive cells after exposure to ST7612AA1 (250 nM) for 72 hrs. The TP53 gene status of each cell line was shown below the X-axis.

We previously identified a highly potent HDAC inhibitor, named ST7612AA1 as prodrug of ST7464AA1 (Figure 1A), showing oral antitumor activity in human tumor-bearing mice. This thioacetyl derivative, selected within a lactam carboxamide inhibitors screening project, showed a high cytotoxic activity on NCI-H460 (NSCLC) and HCT116 (colon carcinoma) cell lines and associated to strong induction of tubulin and histone H4 acetylation in cellular assays [19]. The active drug, ST7464AA1 revealed the maximum potency on HDAC3 and 6 (mean of IC_50_= 4 nM), and then on HDAC1, 10 and 11 (mean of IC_50_=13 nM) and HDAC2 (IC_50_=78 nM). The minor potency was observed on HDAC8 (IC_50_=281 nM) [19].

In this study, the ability of ST7612AA1 in different pre-clinical cancer models characterized by specific protein-overexpression or mutation was determined to better define the pharmacological profile of the drug. Here we report that this novel HDAC inhibitor potently inhibited cell growth/proliferation in human tumor cell lines from both solid and hematologic origin, and significantly suppressed tumor growth in several xenograft models after oral daily delivery, thus suggesting a putative application against some tumor subsets in patients. Furthermore, the drug-dependent modulation of some transcripts involved in immune response and in key pathogenetic pathways, such NF-κB pathway and epithelial-mesenchymal transition, would suggest a relevant implication not only in cancer therapy but also in the inflammatory diseases.

## RESULTS

### ST7612AA1 reduces HDACs activity

We have previously shown that ST7464AA1 (the active drug of ST7612AA1) is a very potent HDAC inhibitor, displaying activity against different HDAC isoforms in the low nanomolar range [19]. Here, we assessed the ability of ST7612AA1 to affect *in vitro* acetylation of tubulin and histone H4 substrates, which is mainly dependent on HDAC6 and class I HDACs respectively, through Western Blot analysis on NCI-H460 NSCLC cells. As shown in Figure 1B, ST7612AA1 was 40-fold more potent in increasing the acetylation of histone H4 (IC_50_ = 4.8 nM) than of tubulin (IC_50_ = 200 nM). Results and details of the densitometry analysis are shown in Supplementary Figure 1. ST7612AA1 was very effective at increasing histone acetylation at concentrations lower than those determining cytotoxicity on the same cell line, thus confirming the ability of its drug (once released within the cell) to bind with a very high affinity to the catalytic site of different HDAC isoforms.

### ST7612AA1 affects proliferation and induces apoptosis in human tumor cell lines

ST7612AA1 showed a high potency in terms of antiproliferative effects in a first broad panel of human tumor cell lines from both solid and hematologic origin. As indicated in Table 1, ST7612AA1 inhibited proliferation in cell lines derived from epithelial cancers (lung, breast, colon, ovarian) and from leukemias and lymphomas, with IC_50_ values ranging from 43 to 500 nmol/L. ST7612AA1 also inhibited the proliferation with comparable potency of different mature B cell lymphomas with a median IC_50_ of 375 nM (range, 46-2664 nM). There were no significant differences among histological subtypes or between germinal center B cell like (GCB) and the activated B cell like (ABC) type –DLBCL: ABC-DLBCL 257 nM (101-805 nM); GCB-DLBCL 597 nM (46-2664 nM); mantle cell lymphoma (MCL) 433 nM (248-553 nM); splenic marginal zone lymphoma (SMZL) 119 nM (102-257 nM). As shown in Supplementary Figure 2, the ST7612AA1 anti-proliferative activity was both time and dose-dependent. Exposure to ST7612AA1 (250 nM) for 72 hrs induced moderate apoptosis in three out of eight lymphoma cell lines (Figure 1C). Differently from what observed regarding on the anti-proliferative activity, the apoptosis was apparently restricted to cell lines bearing a wild type TP53.

**Table 1:** Antiproliferative activity of ST7612AA1 on different human tumor cell lines

Tumor cell line	IC_50_ (μM)
*Ovarian cancer* A2780 SKOV-3	0.043±0.01 0.38±0.003
*Breast cancer* MDA-MB436 MDA-MB231 MCF-7	0.18±0.01 0.23±0.009 0.19±0.02
*NSCLC* NCI-H460 NCI-H1975	0.066±0.01 0.50±0.05
*Colon cancer* HCT116	0.075±0.01
*Acute myeloid leukemia* MV4;11 U937	0.19±0.02 0.049±0.005
*T-cell lymphoma* HUT78	0.48±0.02
*Chronic myeloid leukemia* K562	0.19±0.02
*Diffuse large B-cell lymphoma of the germinal center B-cell type* DOHH2 OCI-Ly8 OCI-Ly7 SUDHL-6 SUDHL-4 VAL Karpas422	0.046±0.01 0.24±0.01 0.56±0.02 0.63±0.02 0.98±0.01 2.36±0.1 2.66±0.2
*Diffuse large B-cell lymphoma of the activated B-cell like type* TMD8 OCI-Ly10 U2932	0.10±0.03 0.26±0.01 0.80±0.02
S*plenic marginal zone lymphoma* K1718 VL51 SSK41	0.10±0.01 0.12±0.09 0.26±0.05
*Mantle cell lymphoma* Jeko-1 MAVER1 Granta-519 REC-1	0.25±0.01 0.39±0.02 0.47±0.05 0.55±0.02

Tumor cells were treated for 72 h at different concentrations to evaluate IC_50_ values. IC_50_ values were determined using ALLFIT program, a sigmoidal dose response model. Results are the means ± S.E.M. of three independent experiments.

### ST7612AA1 affects key molecular pathways in DLBCL *in vitro* models

**Figure 2:**
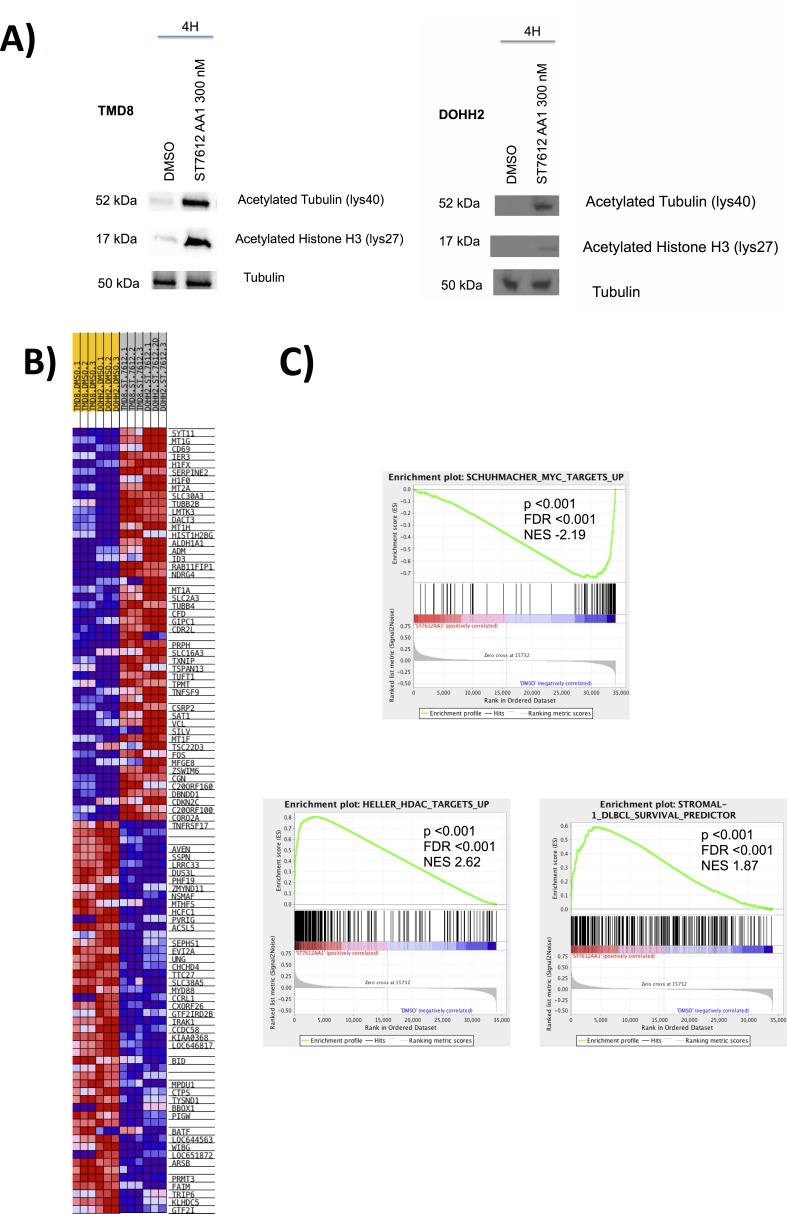
ST7612AA1 affects key molecular pathways in DLBCL A) ST7612AA1 determines acetylation of alpha-tubulin and histone H3 in DOHH2 and TMD8 DLBCL after 4 h exposure. To control for equal loading, blots were probed with antibodies against tubulin. B) Heat map of the top 50 up- top 50 down-regulated rank ordered genes according to GSEA in DOHH2 and TMD8 DLBCL cells exposed to ST7612AA1 (300 nM) for 8 hrs. Expression values are represented as colors, where the range of colors (red, pink, light blue, dark blue) shows the range of expression values (high, moderate, low, lowest). C) GSEA plot illustrating the enrichment of different biologically relevant gene-sets in DOHH2 and TMD8 DLBCL cells exposed to ST7612AA1 as above. FDR, false discovery rate; NES, normalized enrichment score.

To obtain a global view of the transcriptional changes after ST7612AA1 treatment, we performed GEP (Gene Expression Profiling) on two sensitive cell lines, one derived from GCB-DLBCL (DOHH2) and one from ABC-DLBCL (TMD8). We first confirmed the anti-deacetylase activity of ST7612AA1 in the two cell lines (Figure 2A). Then, the tumor cells were exposed to DMSO or to ST7612AA1 (300 nM) for 8 hours. (Figure 2B). ST7612AA1 importantly affected the gene expression profile of the two DLBCL cell lines: applying stringent criteria (genes showing fold change > 1.5, with an adjusted p-value < 0.005, were considered as differentially expressed) 674 genes were up-regulated and 563 down-regulated (Supplementary Table 4). Among the most down-regulated genes there were genes known as oncogenes or involved in lymphoma pathogenesis such as *IRAK1*, *MYD88*, *MYC*, *MYB*, *CCND2*, *BLK*, *CDK4*, *IKZF1* or *TNFRSF17* (*BCMA*). Conversely, the up-regulated ones comprised tumor suppressor genes (*CDKN2C*, *CDKN1A*, *CDKN2D*) or genes involved in immune response (*HLA*, *CD69*). Validation of GEP results was obtained by real-time PCR analysis, confirming the up-regulation of *CDKN1A* and down-regulation of *MYC*, *IRAK4*, *MYD88*, *STAT3*, and, in the ABC-DLBCL cell line, also of *IRAK1* (Supplementary Figure 3). Further insights on the pathways affected by exposure to the HDACi were provided by applying the GSEA algorithm (Supplementary Table 5). Functional analysis highlighted that the down-regulated genes were significantly enriched of MYC targets, E2F targets, transcripts coding proteins involved in cell cycle, RNA processing, G1/S transition, DNA damage checkpoint, genes down-regulated in hypoxia, by other HDACis, or by mTOR inhibitor rapamycin. Up-regulated genes were significantly enriched of genes involved in packaging of telomeres, in meiosis, in RNA polymerase I promoter opening, in autophagy regulation, genes coding components of lysosome, cell adhesion molecules, genes up-regulated by other HDACi, genes of the DLBCL prognostically favorable stromal signature. Figure 2C shows some of the gene sets significantly enriched among down- and up-regulated genes.

### ST7612AA1 causes growth inhibition of different tumor xenografts

Following the observed potent *in vitro* inhibition of tumor cell proliferation by ST7612AA1, we subsequently investigated whether these properties translated into tumor growth inhibition in preclinical *in vivo* models. Oral ST7612AA1 (60 mg/10 mL/kg, qdx5/w, for 2-4 weeks), strongly inhibited the growth of different pre-established tumor xenografts. In particular, as shown in Table 2, ST7612AA1 inhibited tumor volume by 77% (*P*<0.01 vs vehicle treated group) in the colon carcinoma model HCT116, consistently with the antiproliferative effect achieved *in vitro* against the same tumor cell line. Analogously, a potent and significant antitumor activity of ST7612AA1 was also shown against other solid tumor xenografts, such as the NSCLC model NCI-H1975 (TVI=65%, *P*<0.001), the ovarian carcinoma model SKOV-3 (TVI=59%, *P*<0.01) and the breast cancer model MDA-MB436 orthotopically implanted in mammary fat pad (TVI=35%, P<0.05). Finally, *in vivo* antitumor efficacy of ST7612AA1 was also observed against hematological tumor models, as shown by the potent antitumor activity (TVI=70%, *P*<0.01) in the AML model MV4;11 (Table 2) and in the GCB-DLBCL model DOHH2 bearing both MYC and BCL2 chromosomal rearrangement in which a significant delay in tumor progression (*P*<0.05) was observed (Supplementary Figure 4).

**Table 2:** Antitumor activity of ST7612AA1 against different human tumor cell xenografts in nude mice

Tumor cells	DT	Treatment schedule	TVI%	BWL% max	Lethal toxicity
*Colon cancer* HCT-116	5.9	Qdx5/wx3w (3-7, 10-14, 17-21)	**77	1	0/8
*NSCLC* NCI-H1975	3.5	Qdx5/wx2w (5-9, 12-16)	***65	4	0/8
*Ovarian cancer* SKOV-3	7.9	Qdx5/wx4w (3-7, 10-14, 17-21, 24-28)	**59	1	0/8
*Breast cancer* MDA-MB436	9.5	Qdx5/wx4w (6-10, 13-17, 20-24, 27-31)	*35	4	0/8
*Acute myeloid leukemia* MV4;11	6.6	Qdx5/wx3w (11-15, 18-22, 25-29)	**70	4	0/8

Mice bearing subcutaneously implanted tumor cells were orally administered with ST7612AA1 (60 mg/10 mL/kg) every day according to a schedule Qdx5/w. TVI was calculated 8-10 days after the end of treatment. n=8 mice/group.

DT, Doubling Time. TVI, tumor volume inhibition. BWL%, maximum body weight loss during the experimental period.

Lethal toxicity: Number of mice dead from toxicity/total number of mice. The statistic comparison was performed between the mean of tumor lesions of drug-treated group and the mean of tumors of vehicle-treated group.

* P<0.05 vs vehicle (Mann-Whitney test).

** P<0.01 vs vehicle (Mann-Whitney test).

*** P<0.001 vs vehicle (Mann-Whitney test).

### ST7612AA1 affects key molecular pathways in colon cancer *in vivo* model

**Figure 3:**
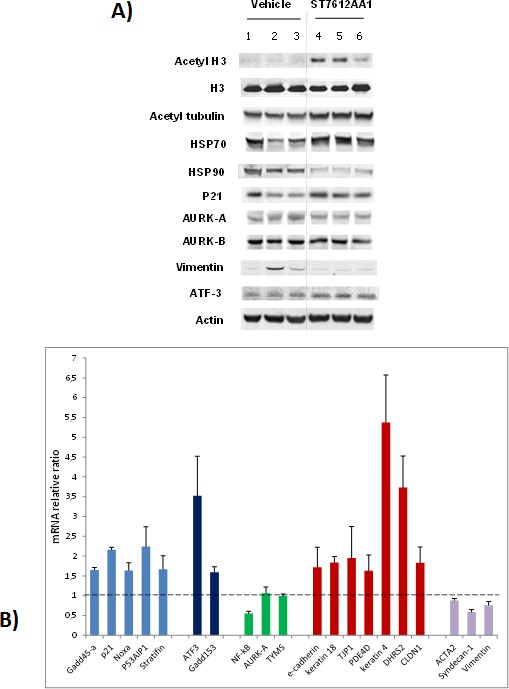
Effect of ST7612AA1 on key molecular targets in colon cancer A) Western Blot analysis for assessing the degree of acetylation of histone H3 and tubulin, and for evaluating the expression levels of various target proteins in HCT-116 tumor xenografts collected 24 hours after the last treatment with 80 mg/10 mL/kg ST7612AA1 (lanes 4-6) once daily, according to the schedule qdx5/wx3w, with respect to vehicle-treated animals (lanes 1-3). Actin is shown as a control for protein loading. Representative blots of tumor samples from 3 animals/group are shown. B) Real-time qPCR analysis of ST7612-induced gene changes in HCT-116 tumor xenografts collected as above described. Data are normalized to cyclophilin A and presented as fold change (average ± s.d.) over the vehicle-treated control mice (n=3 animals/group). Sybr Green-based q-PCR analysis was performed using the primer set shown in Suppl. Table 3.

Western Blot analysis of the HCT116 tumor xenografts collected 24 h after the last oral administration of ST7612AA1 revealed a strong induction of pan H3 acetylation (Figure 3A). Besides restoring histone acetylation through inhibition of class I HDACs, ST7612AA1 was also effective in targeting HDAC6, as shown by the increased levels of acetylated α-tubulin and by the dramatic decrease of HSP90 protein levels (this effect likely due to hyperacetylation of the chaperone), paralleled by a significant increase of HSP70 levels. Moreover, treatment of HCT116 tumor-bearing mice with ST7612AA1 resulted in up-regulation of P21 and ATF3 proteins, also confirmed at the transcriptional level in Figure 3B, thus suggesting that molecular pathways, activated by DNA damage events (TP53-mediated or not), or associated to a putative ER-stress response, might be involved. This evidence is further supported by qPCR data, showing increased mRNA levels of several genes associated to DNA-damage (*gadd45α*), TP53-mediated pro-apoptotic events (*Noxa*, *P53AIP1*, *stratifin*) and ER-stress response (*gadd153/CHOP*). Treatment with ST7612AA1 also resulted in down-modulation of NF-κB gene (Figure 3B), whereas no effect was observed on the expression of genes involved in DNA replication, such as *TYMS* and *AURK-A*, although the last one was moderately down-modulated at the protein level (Figure 3A). Finally, because HDACis have been shown to counteract the EMT process in different tumor models [20, 21], we next assessed the effects of ST7612AA1 on epithelial/mesenchymal markers in HCT116 tumor xenografts. As shown in Figure 3B, ST7612AA1 induced a significant overexpression of several genes (*e-cadherin*, *keratins 4/18*, *TJP1*, *PDE4D*, *claudin 1*) coding typical epithelial markers and a concomitant down-modulation of genes associated to mesenchymal phenotype, such as *ACTA2*, *syndecan-1* and *vimentin* (the last one being dramatically down-regulated also at the protein level, as depicted in Figure 3A). Overall, biochemical data suggest that reversion of the EMT process might contribute to the *in vivo* antitumor activity of the drug.

### ST7612AA1 is *in vivo* rapidly converted to ST7464AA1

**Figure 4:**
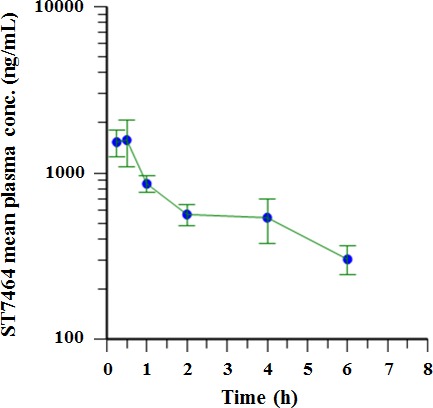
Plasma concentration-time profile of ST7464AA1 following oral (PO) administration of ST7612AA1 to mice The pharmacokinetic parameters of ST7464AA1 are shown in Table 3. Mean (± SEM) plasma concentration versus time of ST7464AA1 after a single oral dose of 120 mg/kg of ST7612AA1 in CD1 male mice (lin-log scale) (n=5).

The PK profile of ST7612AA1 (pro-drug) and ST7464AA1 (drug) in healthy mice was determined after a single dose of 120 mg/kg of compound administered by oral route. As expected, ST7612AA1 was not detected in plasma after oral administration in mice, confirming its pro-drug properties. Conversely, ST7464AA1 rapidly appeared in plasma being quantifiable at the first blood sampling time (0.25 h). ST7464AA1 reached the C_max_ of 1577 ± 478 ng/mL (as mean ± SEM) 0.5 h post dosing; then its plasma concentration declined according to a bi-exponential profile (Figure 4) being still quantifiable at the last blood sampling time (6 h). ST7464AA1 was cleared from plasma with a T_1/2_ of 3.8 h. ST7464AA1 pharmacokinetics parameters are summarized in Table 3. The drug showed a high CL/F; furthermore, its large Vz/F indicated a good propensity to distribute outside the systemic circulation.

**Table 3:** Pharmacokinetic parameters for ST7464AA1 in CD1 mice receiving a single oral dose of 120 mg/kg of ST7612AA1 derived from the plasma concentration vs time data according to a model independent approach for sparse data sampling

T_max_ (h)	C_max_* (ng/mL)	T_last_ (h)	C_last_ (ng/mL)	AUC_last_* (h* ng/mL)	AUC_INF_ (h*ng/mL)	CL/F (mL/h/kg)	Vz/F (mL/kg)	T_1/2_ (h)
0.5	1577±478	6.0	303	3747±299	5506	21795	119340	3.8

C_max_: maximum plasma concentration; T_max_: time of C_max_; C_last_: last quantifiable concentration; T_last_: time of C_last_; AUC_last_: (area under the concentration vs time curve from 0 to T_last_; AUC_INF_: area under the concentration vs time curve from 0 to infinity; T_1/2_: terminal half-life; CL/F: apparent systemic clearance; Vz/F: apparent terminal volume of distribution

* mean ± SEM.

## DISCUSSION

Recently, a systematic study of medicinal chemistry aimed at identifying a new generation of HDAC inhibitors led us to select a new class of thiol-based potent pan-HDACis [19]. *In vivo* pharmacodynamic analysis of several preselected analogues resulted in the identification of ST7612AA1, (property of Sigma-Tau), a thioacetate-ω (γ-lactam amide) derivative, first synthetic thiol derivative, as potent oral pan-histone deacetylase inhibitor (HDACi), in preclinical phase. This a prodrug of ST7464AA1, which has exquisite potency toward all class I HDACs (IC_50_ values of 12.7 and 77.7 nM for HDAC1 and HDAC2, respectively) and toward HDAC isoforms encompassed within the class IIb HDACs (IC_50_ value of 3.18 nM for HDAC6). In agreement with the powerful inhibition of class I HDACs, here, we show that ST7612AA1 had a broad spectrum antiproliferative activity on cell lines derived from ovarian cancer, breast cancer, NSCLC, colon cancer and haematological tumors, including acute monocytic leukaemia, chronic myeloid leukaemia and lymphoma, at concentrations that are significantly below those achieved in plasma of mice (Cmax, 1577 ± 478 ng/mL, dosing ST7612AA1 po at 120 mg/kg). Importantly, the oral treatment with ST7612AA1, once daily, for 2 or 3 weeks, strongly inhibited tumor growth in several preclinical *in vivo* models derived from both solid tumors and haematological cancers. In particular, ST7612AA1 was able to significantly inhibit tumor growth of the Ras-mutant HCT116 colon carcinoma xenografts, thus suggesting a putative therapeutic approach towards this subset of strongly proliferating dedifferentiated colorectal carcinoma, characterized by overexpression of class I HDAC family members and associated with reduced patient survival [22].

In addition, our data indicate that ST7612AA1 can significantly inhibit *in vitro* the proliferation of NSCLC cell lines bearing wild type EGFR (and mutant KRAS), such as NCI-H460, as well as a secondary (T790M) EGFR mutation, which is known to confer resistance to tyrosine kinase inhibitors [23], such as NCI-H1975. Interestingly, ST7612AA1 showed also a significant *in vivo* antitumor effect against the latter tumor xenograft model.

Since class I HDAC isoforms are expressed at significantly higher levels in ovarian cancer compared to normal ovarian tissue [24], and various HDAC inhibitors can prevent both *in vitro* and *in vivo* growth of ovarian cancer cells [25, 26], we investigated the *in vivo* efficacy of ST7612AA1 also in two ovarian carcinoma models. Our data clearly showed a strong antitumor effect of ST7612AA1 against both the SKOV-3 model, characterized by low levels of PTEN and overexpression of EGFR and ErbB2 [27], and even more the A2780 xenograft, characterized by the absence of PTEN [19].

Triple-negative breast cancer (TNBC) represents a heterogeneous subset of neoplasms defined by the absence of estrogen receptor (ER), progesterone receptor (PR) and Her2/neu, which accounts for approximately 15% of globally diagnosed breast cancers and which does not respond to hormonal therapy (such as tamoxifen or aromatase inhibitors) or therapies that target HER2 receptors [28-31]. Anyhow, recent papers have suggested a putative therapeutic approach also with HDAC inhibitors [32, 33]. ST7612AA1 evidenced a strong antiproliferative activity *in vitro* against two TNBC cell lines (MDA-MB231 and MDA-MB436). Moreover, when tested *in vivo* against the BRCA1-defective MDA-MB436 tumor xenograft ortotopically implanted in mammary fat pad, ST7612AA1 caused a significant reduction of tumor growth associated to minimal animal toxicity, thus providing promising preclinical data that would suggest a putative therapeutic approach against this subset of breast cancer.

The ST7612AA1 treatment resulted also in a significant tumor growth inhibition of the AML model MV4;11, subcutaneously implanted in athymic nude mice. MV4;11 tumor is known to be driven by the tyrosine kinase receptor Flt3-ITD mutation. The activating internal tandem duplications (ITD) in the juxtamembrane domain of FLT3 have been identified in 35% AML patients [34]. MV4;11 has been shown to be dependent on FLT3-ITD by its sensitivity to selective FLT3 kinase inhibitors [35]. The best approach to the treatment of FLT3-ITD AML is currently undefined, and multiple clinical trials are investigating FLT3 kinase inhibitors [36] but, unfortunately, their action is very often transient, possibly due to inadequate dosing or insufficient selectivity of these drugs. For these reasons, treatment with our HDACi might represent a promising therapeutic option also for patients with this kind of tumor.

Deregulation of proteins involved in chromatin remodelling is very frequent in lymphomas, which represent an interesting target for HDACi [12, 13, 37]. Here, ST7612AA1 presented a wide *in vitro* anti-proliferative activity on various models of lymphomas, induced apoptosis in TP53 wild type lymphoma cells, affected relevant pathogenetic pathways in DLBCL cell lines, and also reduced the growth of DLBCL xenografts. In particular, ST7612AA1 affected the NF-κB signaling, and this is of particular interest for the important role played by this pathway in the pathogenesis of certain lymphoma subtypes, such as the ABC-DLBCL, MCL and marginal zone lymphomas [38, 39]. Moreover, the compound was also able to down-regulate MYC target genes, and this might be clinically relevant for DLBCL, in which MYC confers a very poor clinical outcome when co-expressed with the BCL2 protein or when co-translocated with the *BCL2* gene [39, 40]. Importantly, the *in vivo* antitumor activity of ST7612AA1 was indeed observed against a cell line characterized by both MYC and BCL2 gene translocations. Thus, the compound appears worth of further investigation in the lymphoma context. Furthermore, present molecular and biochemical data suggest that, once hydrolyzed, ST7612AA1 acts both in nucleus and cytoplasm of the target tumor cell, through HDAC6 inhibition, as observed for other HDACi of the hydroxamate class [41]. In fact, beside restoring the balance of the histone acetylation that, in turn, results in a more relaxed chromatin structure, with areas of loosely compacted, and hencemore transcriptionally active chromatin that is more prone to DNA double strand breaks [42], ST7612AA1 is also able to target non-histone HDAC substrates involved, for example in the regulation of multiple cellular functions, such as P53, *alpha*-tubulin or the heat shock protein 90 (HSP90), through inhibition of HDAC6, which has been implicated in DNA damage signaling, transcription factor binding, and DNA repair processes [43]. Interestingly, at least when tested *in vivo* against colon carcinoma xenografts, ST7612AA1 induced increased transcription of e-cadherin, keratins and other typical epithelial markers and, concomitantly, induced down-regulation of vimentin and other genes associated to the mesenchymal phenotype, thus suggesting that treatment with ST7612AA1 might also cause a “cadherin switch” and reversion of the EMT process. Other HDAC inhibitors such as SAHA, TSA and panobinostat were shown to induce EMT phenotype, which was associated with increased expression of mesenchymal markers such as vimentin, N-cadherin and fibronectin [44, 45]. The ability of cells to transdifferentiate and dedifferentiate plays a key role in invasion and metastasis by the process of epithelial-mesenchymal-transition (EMT) [46], and differentiation patterning may be used as an additional prognostic and predictive indicator for therapeutic effectiveness.

Histone deacetylase inhibitors (HDACi) were primarily developed as anti-tumor agents for cancer, but many are now being explored for treating neurodegenerative, immunologic, metabolic, inflammatory and cardiovascular disorders [47].

ST7612AA1 was able to determine expression changes of transcripts involved in immune response and in key pathogenetic pathways, such as the NF-κB pathway and cell cycle alteration, thus suggesting a relevant putative involvement not only in cancer therapy but also in the inflammatory diseases [48]. Lysine acetylation is a key regulator of the NF-κB pathway, which works in concert with other PTMs *via* complex crosstalk mechanisms to determine the signaling output. Importantly, small molecule modulators of its writers (HATs) or erasers (HDACs) have been demonstrated to regulate NF-κB signaling, suggesting that these are potential drugs for inflammatory diseases.

These data combined with the excellent *in vivo* tolerability and the oral delivery may represent a therapeutic advantage for this novel HDAC inhibitor.

In conclusion, based upon the obtained data, ST7612AA1 appears as a candidate for clinical development to evaluate its therapeutic activity towards a broad spectrum of human solid and haematologic malignancies.

## MATERIALS AND METHODS

### Ethics statement

All animal experiments were conducted according to relevant national and international guidelines. Experimental protocols were approved by the Ethic Committee for Animal Experimentation of Sigma Tau according to the United Kingdom Coordinating Committee on Cancer Research Guidelines. When tumor volume exceeded 2 cm^3^, mice were euthanized by cervical dislocation.

### Drugs

For *in vitro* experiments, stock solutions of ST7612AA1 (property of Sigma-Tau) were prepared in 100% dimethyl sulfoxide at 10 mM and stored at −20°C. For oral administration, ST7612AA1 was dissolved in solutol HS15 + water (1:20) and delivered in a volume of 10 mL/kg.

### Cell lines and cell culture

Supplementary Table 1 lists all the cell lines used with their growth conditions and their origin. All cells were maintained in a humidified atmosphere with 5% CO_2_ at 37°C. All experiments were always performed starting from frozen cell stocks of each cell line. Upon thawing, such cells were characterized in house, by assessing cell morphology, cell growth kinetics curve and absence of mycoplasma. The human cell lines purchased from accredited biological resource centers (i.e. ATCC, ECACC, DSMZ) have been originally authenticated and characterized directly by the providers (STR profiling). Lymphoma cell lines were validated by the Authors using DNA profiling within the last six months from the beginning of the study. All the experiments have been then performed using cells within 6-8 passages since thawing from an internal cell bank.

### *In vitro* proliferation assay

Anti-proliferative activity was first assessed on a panel of cell lines derived from solid tumors and hematological cancers and then on a large panel of cell lines derived from mature B-cell lymphomas. In the first panel, cells were seeded in 96-wells tissue culture plates in complete medium and, 24 h after seeding, were exposed to increasing concentrations of ST7612AA1 for 72 h; the inhibition of proliferation was assessed by the sulphorodamine B assay. Lymphoma cells were seeded in 96-wells tissue culture plates in complete medium and were exposed to increasing concentrations of ST7612AA1 for 48 or 72 h; the anti-proliferative activity was assessed using 3-(4,5-dimethylthiazol-2-yl)-2,5-diphenyltetrazolium bromide (MTT). The drug potency was evaluated by means of the “ALLFIT” computer program and defined as IC_50_ (drug concentration required for 50% inhibition of cell survival).

### Detection of apoptotic cells

Apoptosis was assessed, on cells treated with DMSO or different doses of ST7612AA1, by Annexin V-FITC apoptosis detection kit (BD Biosciences), according to the manufacturer's recommendations, on a FACScan flow cytometer (BD Biosciences).

### Western blotting analysis

For assessing the *in vitro* effect of ST7612AA1 on acetylation of α-tubulin and histones, NSCLC cells were treated with the test compound at various concentrations (dose-response curve). SAHA 5 μM was used as reference inhibitor. Protein extraction, separation and immunoblotting were performed as previously described [19]. Immunoreactive bands were finally subjected to densitometry analysis by a phosphoimaging system (STORM, Molecular Dynamics), and then the IC_50_ values were calculated by the “ALLFIT” computer program. The antibodies used are listed in Supplementary Table 2.

### *In vivo* xenograft models

Experiments on solid tumor and acute leukemia *in vivo* models were carried out at Sigma-Tau (Rome, Italy) using female athymic nude mice, 5-6 weeks-old (Harlan Laboratories, Udine, IT). Mice were maintained in laminar flow rooms with constant temperature and humidity in according to the NIH guidelines. The following human tumor xenograft models were used for antitumor activity studies: HCT116 derived from colon carcinoma), MV4;11 from acute myeloid leukaemia (AML), SKOV-3 from ovarian carcinoma, and NCI-H1975 from non-small cell lung cancer (NSCLC). Exponentially growing tumor cells were s.c. inoculated (5×10^6^/mouse) in the right flank of nude mice. Groups of eight mice/group were employed to assess antitumor activity. MDA-MB436 breast carcinoma (3×10^6^ cells/0.1 mL of M199/Matrigel GFR 50:50, vol/vol solution) was inoculated in mammary fat pad (mfp). Drug treatments were started from 3 to 11 days after tumor injection, depending on the tumor growth of the xenografted cancer model. ST7612AA1 was given daily for five days per week (qdx5/w). Tumor growth was followed by measurements of tumor diameters with a Vernier caliper. Tumor volume (TV) was calculated using the formula: TV (mm^3^) = [d^2^ x D]/2, where d and D are the shortest and the longest diameter, respectively. The efficacy of the drug treatment was assessed as: TV inhibition percentage (TVI%) in treated versus control mice, calculated as: TVI%=100-(mean TV treated/mean TV control x100). When tumors reached a volume of 500-1000 mm^3^, mice were sacrificed by cervical dislocation. To examine the possible toxicity of treatment, body weight was recorded throughout the study. BWL% (body weight loss) was calculated as 100 – (mean BW_dayx_/mean BW_day1_x100), where day 1 is the first day of treatment and day x is any day after (maximum BWL%). DT as doubling time of control tumors was also evaluated.

In order to assess the *in vivo* effect of ST7612AA1 on the acetylation degree of α-tubulin and histones, and on the expression of other key proteins, HCT116 tumor xenografts (3 samples/group) were excised at different times after the last treatment, and then total protein lysates were prepared through the homogenization of tumor samples in lysis buffer containing 0.5% NP-40, supplemented with 10 μg/mL of protease inhibitor cocktail (Sigma Chemical Co., St. Louis, MO, USA). Determination of the protein concentration and Western Blotting analysis were finally performed as above described for the *in vitro* experiments. The antibodies used are listed in Supplementary Table 2.

The *in vivo* experiment with lymphoma model was performed in the IOR laboratory according to study protocols approved by the local Cantonal Veterinary Authority (No. 5/2011). At day 1, tumors were established by injecting DOHH2 lymphoma cells (200 μL of PBS, 8×10^6^ cells/mouse) into the left flanks of 5-weeks old female NOD-SCID mice (Harlan Laboratories). Tumor size was measured on regular basis and until tumors reached around 0.5 mm in diameter (day 12). Then, treatments were conducted at day 12, 13, 15, 16, 18, 19. Tumors were measured at 12, 14, 17, 21, 25 days. Tumor volumes were calculated as described above. Mice were sacrificed when physical conditions became critical or when tumors reached a weight of about 0.5 gr. For comparison between a control and a treatment group, an unpaired Mann-Whitney's test was used. A *P*-value <0.05 was considered significant.

### Quantitative real-time RT-PCR

Total RNA was extracted from tumour xenografts and then retrotranscribed using the Trizol reagent and the ThermoScript RT-PCR System (Invitrogen, Paisley, UK), respectively, according to the manufacturer's instructions. SYBR Green-based qPCR analyses were performed in 96-well plates by using the 7900HT Sequence Detection System instrument and software (Applied Biosystems). Amplification mixes (20 μL) contained 1x QuantiTect SYBR PCR kit (QIAGEN, Hilden, Germany), and 0.2-0.3 μM of each specific primer. In addition, the mRNA levels of *cyclophilin A* were quantitatively measured in each sample to control for sample-to-sample differences in RNA concentration. The cycling conditions comprised a 600 s denaturation step at at 95°C, followed by 40 cycles of denaturation at 95°C for 15 s, annealing at 60°C for 20 s, and extension at 72°C for 10 s. The oligonucleotides used as specific primers for each target gene were designed, using the manufacturer's software and the sequences available in GenBank, to overlap a splice junction thereby avoiding a potential amplification of contaminating genomic DNA, and are described in Supplementary Table 3. A six-point serial standard curve was generated for each target gene. All expression levels were finally normalized to *cyclophilin A* in each well.

### PK sampling and analysis

CD1 nude mice were used. Male mice were treated with a single dose of ST7612AA1 at 120 mg/10 mL/kg p.o., using 5% Solutol HS 15 in water for injection as vehicle. Blood samples were collected at 0.25, 0.5, 1, 2, 4 and 6 h post treatment from 5 animals per time point. Levels of ST7612AA1 and ST7464AA1 were determined in plasma by quantitative LC-MS/MS having a limit of quantification of 25 ng/mL. The PK parameters C_max_ (maximum plasma concentration), T_max_ (time of maximum plasma concentration), C_last_ (last quantifiable concentration), T_last_ (time of last quantifiable plasma concentration), AUC_last_ (area under the concentration *vs* time curve from 0 to T_last_), AUC_INF_ (area under the concentration *vs* time curve from 0 to infinity), T_1/2_ (terminal half-life), CL/F (apparent systemic clearance) and Vz/F (apparent terminal volume of distribution) were derived from the analyte plasma concentration vs time data according to a model independent approach for sparse data sampling by using Phoenix WinNonlin^®^ ver. 6.3 software (Pharsight, Cetara).

### Gene expression profiling

Gene Expression Profiling (GEP) was done using the HumanHT-12 v4 Expression BeadChip (Illumina, San Diego, CA, USA), as previously described [49]. Three replicates were done for each condition. Data were quantile normalized and differential expression analysis was performed using LIMMA [50]. Quantitative Real-time Polymerase chain reaction (qRT-PCR) was performed as previously described [49] (primer sequences available upon request). Gene-sets differentially affected by exposure to ST7612AA1 were identified with the Gene Set Enrichment Analysis (GSEA) tool using the GSEA C2, C3.tft, C6 collections [51] and the Signature DB collection [52]. Raw data are available at the National Center for Biotechnology Information (NCBI) Gene Expression Omnibus (GEO) (http://www.ncbi.nlm.nih.gov/geo) database (series record: GSE62460).

### Statistical analysis

Data are expressed as the mean ± S.E.M. Statistical analysis was performed using Mann-Whitney's test. A *P*-value of <0.05 was considered statistically significant.

## SUPPLEMENTARY TABLES AND FIGURES






