# Supplementary material for: Preclinical antitumor activity of ST7612AA1: a new oral thiol-based histone deacetylase (HDAC) inhibitor
Source: Oncotarget. 2014 Dec 25;6(8):5735–48. doi: 10.18632/oncotarget.3240 (PMC4467398; doi:10.18632/oncotarget.3240)

Supplementary Tables and Figures

Supplementary Table 1. List of cell lines with growth conditions and origin**.**

Supplementary Table 2. List of antibodies used for Western Blotting analyses.

Supplementary Table 3. List of primers used for real-time qPCR analyses.

Supplementary Table 4. Gene expression profiling of in DOHH2 and TMD8 DLBCL cells exposed to ST7612AA1 (300 nM) for 8 hrs: results of limma analysis**.** Column A, Illumina probe; Column B, gene; Column C, log ratio between ST7612AA1-treated and DMSO-treated DLBCL cells; Column D, P value between ST7612AA1-treated and DMSO-treated DLBCL cells; Column E, adjusted P value between ST7612AA1-treated and DMSO-treated DLBCL cells; Column F, average expression value in DMSO-treated DOOH2 and TMD8 cells; Column G, average expression value in ST7612AA1-treated DOOH2 and TMD8 cells.

Supplementary Table 5. Gene expression profiling of in DOHH2 and TMD8 DLBCL cells exposed to ST7612AA1 (300 nM) for 8 hrs: results of GSEA analysis with up to the ten most significant gene sets for each GSEA dataset. A, Enrichments among the down-regulated genes after ST7612AA1 exposure. B, Enrichments among the up-regulated genes after ST7612AA1 exposure. In each table: Column A, GSEA collection; Column B, GSEA geneset name; Column C, Normalized Enrichment Score; Column D, nominal p-value; Column E, FDR, false discovery rate.

Supplementary Figure 1. Densitometric analysis of H4 and tubulin acetylation after treatment with ST7612AA1. ST7612AA1 showed to increase H4 and tubulin acetylation.

Supplementary Figure 2. ST7612AA1 inhibited the tumor cell growth of DLBCL at different times (48 or 72h).

Supplementary Figure 3. Up or down-regulation of some genes obtained by real-type qPCR analysis was used for GEP (Gene Expression Profiling) validation results.

Supplementary Figure 4. ST7612AA1 decreased tumor volumes in the lymphoma DOHH2 model. Treated mice with ST7612AA1 po at days 12, 13, 15, 16, 18, 19 after tumor injection, were compared with control mice. ST7612AA1 resulted significantly active at day 14 (P=0.044), after two administrations, and at day 17 (P=0.045), after four administrations.

Supplementary Table 1. List of cell lines with growth conditions and origin.

Study phase	Histology	Cell line	Growth Medium*	Origin
First panel				
	Breast carcinoma	MDA-MB231	RPMI-1640 **	ATCC
	Breast carcinoma	MDA-MB436	RPMI-1640 **	ATCC
	Colorectal cancer	HCT116	McCoy's 5A **	Istituto Zooprofilattico, Brescia, IT
	Non-small cell lung carcinoma	NCI-H1975	RPMI-1640 **	ATCC
	Non-small cell lung carcinoma	NCI-H460	RPMI-1640 **	ATCC
	Ovarian cancer	A2780	RPMI-1640 **	ECACC
	Ovarian cancer	SKOV-3	RPMI-1640 **	ATCC
	Acute myeloid leukemia	MV4;11	IMDM **	ATCC
	Acute monocytic leukemia	U937	RPMI-1640 **	ATCC
	Chronic myeloid leukemia	K562	RPMI-1640 **	ATCC
Second Panel				
	Cutaneous T-cell lymphoma	HUT78	RPMI-1640 **	ATCC
	Diffuse large B-cell lymphoma of the activated B-cell like type	OCI-Ly10	IMDM ***	kindly provided by Laura Pasqualucci, New York, NY, USA
	Diffuse large B-cell lymphoma of the activated B-cell like type	TMD8	RPMI-1640 ***	kindly provided by Fabio Martinon, Lausanne, CH
	Diffuse large B-cell lymphoma of the activated B-cell like type	U2932	RPMI-1640 ***	kindly provided by Bettina Borisch, Geneva, CH
	Diffuse large B-cell lymphoma of the germinal center B-cell type	DOHH2	RPMI-1640 ***	kindly provided by Finbarr Cotter, London UK
	Diffuse large B-cell lymphoma of the germinal center B-cell type	Karpas422	RPMI-1640 ***	DSMZ
	Diffuse large B-cell lymphoma of the germinal center B-cell type	OCI-Ly7	RPMI-1640 ***	kindly provided by Laura Pasqualucci, New York, NY, USA
	Diffuse large B-cell lymphoma of the germinal center B-cell type	OCI-Ly8	RPMI-1640 ***	kindly provided by Laura Pasqualucci, New York, NY, USA
	Diffuse large B-cell lymphoma of the germinal center B-cell type	SU-DHL-4	RPMI-1640 ***	kindly provided by Gianluca Gaidano, Novara, IT
	Diffuse large B-cell lymphoma of the germinal center B-cell type	SU-DHL-6	RPMI-1640 ***	kindly provided by Louis M. Staudt, Washington, DC, USA
	Diffuse large B-cell lymphoma of the germinal center B-cell type	VAL	RPMI-1640 ***	kindly provided by José Ángel Martinez-Climent, Pamplona, SP
	Mantle cell lymphoma	Granta-519	DMDM ***	DSMZ
	Mantle cell lymphoma	Jeko-1	RPMI-1640 ***	kindly provided by Eisaku Kondo, Okayama, Japan
	Mantle cell lymphoma	MAVER1	RPMI-1640 ***	kindly provided by Alberto Zamo', Verona, IT
	Mantle cell lymphoma	REC-1	RPMI-1640 ***	kindly provided by Finbarr Cotter, London UK
	Splenic marginal zone lymphoma	Karpas-1718	RPMI-1640 ***	kindly provided by José Ángel Martinez-Climent, Pamplona, SP
	Splenic marginal zone lymphoma	SSK41	RPMI-1640 ***	kindly provided by José Ángel Martinez-Climent,

			Pamplona, SP
Splenic marginal zone lymphoma	VL51	RPMI-1640 ***	kindly provided by José Ángel Martinez-Climent, Pamplona, SP

ATCC, American Type Culture Collection (Manassas, VA, U.S.A); DSMZ, Deutsche Sammlung von Mikroorganismen und Zellkulturen (Braunschweig, Germany); ECACC, European Collection of Animal Cell Cultures (Porton Down, Salisbury, United Kingdom)

*All media were supplemented with fetal calf serum (10% or 20%), Penicillin-Streptomycin-Neomycin (~5,000 units penicillin, 5 mg streptomycin and 10 mg neomycin/mL, Sigma) and L-glutamine (1%); ** Lonza, Verviers, Belgium; ***, GIBCO Invitrogen, Basel, Switzerland;

Supplementary Table 2.Antibodies used for Western Blotting analyses

Target protein	Antibody
Acetyl-histone H3	Rabbit anti-acetyl-histone H3 (Lys9/Lys14) (cat. 9677) – Cell Signaling
Acetyl-histone H4	Rabbit anti-acetyl-histone H4 (cat. 06-598) – Merck Millipore
Acetyl-tubulin	Mouse anti-acetyl-α tubulin (clone 6-11-B-1) (cat. T6793) - Sigma
Actin	Mouse anti-b-actin (clone AC-74) (cat. A5316) - Sigma
ATF-3	Rabbit anti-ATF3 (C-19) (cat. sc-188) – Santa Cruz Biot.
Aurora kinase A	Rabbit anti-AURK-A (cat. 1800-1) - Epitomics
Aurora kinase B	Rabbit anti-AURK-B (cat. 1788-1) - Epitomics
Histone H3	Rabbit anti-histone H3 (cat. NB500-171) – Novus Biol.
Histone H4	Rabbit anti-histone H4 (cat. 07-108) – Merck Millipore
HSP70	Mouse anti-Hsp70 (BRM-22) (cat. H5147) - Sigma
HSP90	Mouse anti-Hsp90 (4F10) (cat. sc-69703) - Santa Cruz Biot.
P21	Rabbit anti-p21 (C-19) (cat. sc-397) - Santa Cruz Biot.
α-Tubulin	Mouse anti-α-Tubulin (clone B-5-1-2) (cat. T5168) - Sigma
Vimentin	Rabbit anti-Vimentin (cat. ab45939) - abcam

Supplementary Table 3. Primers used for Real-Time qPCR.

GeneBank accession n°	Gene	Sequence
NM_001141945	ACTA2	Forward: 5'- CCGCCCCACTTTGCCTAT-3'<br>Reverse: 5'- TGGAGCTGCTTCACAGGATTC-3'
NM_001674	ATF3	Forward: 5'- CCAGCAGCAGAGAACCATCAA-3'<br>Reverse: 5'- AAAGATGCACTTGCCCTGGTC-3'
NM_198433	AURK-A	Forward: 5'- GGTGGCTCATGCCCGTAA-3'<br>Reverse: 5'- GGTTGCAGTGAGCTGAGATCAC-3'
NM_021101	CLDN1	Forward: 5'- GCACCGGGCAGATCCA-3'<br>Reverse: 5'- TTGCAATGTGCTGCTCAGATT-3'
NM_021130	Cyclophilin A	Forward: 5'- ACAAGGTCCCAAAGACAGCAGA-3'<br>Reverse: 5'- CCCTGACACATAAACCCTGGAA-3'
NM_182908	DHRS2	Forward: 5'- TCTGGGACAAGATCCTAAGTGTGA-3'<br>Reverse: 5'- TCCATGTAGGGCAGCAACTG-3'
NM_004360	e-cadherin	Forward: 5'- ACAGCCCCGCCTTATGATT-3'<br>Reverse: 5'- TCGGAACCGCTTCCTTCA-3'
NM_001924	gadd45-α	Forward: 5'- ACGGTGATGGCATCTGAATGA-3'<br>Reverse: 5'- CCCCTTGGCATCAGTTTCTGT-3'
NM_004083	gadd153 (CHOP)	Forward: 5'- AACCAGCAGAGGTCACAAGCAC-3'<br>Reverse:5'-TCCTGGTTCTCCCTTGGTCTTC-3'
NM_002272	Keratin 4	Forward: 5'- CACCTCCAGCAAAAAACCTTGA-3'<br>Reverse: 5'- TCTAGCTGCTTCCTCAGGACACT-3'
NM_000224	Keratin 18	Forward: 5'- GCGAGGACTTTAATCTTGGTGATG-3'<br>Reverse: 5'- TGGTCTTTTGGATGGTTTGCA-3'
NM_003998	NF-KB	Forward: 5'- GGCTACACCGAAGCAATTGAA-3'<br>Reverse: 5'- CAGCGAGTGGGCCTGAGA-3'
NM_021127	Noxa	Forward: 5'- ACAAACTGAACTTCCGGCAGA-3'<br>Reverse: 5'- TTTGAAGGAGTCCCCTCATGC-3'
NM_000389	P21	Forward: 5'- CCTCCCCAGTTCATTGCACTT-3'<br>Reverse: 5'- AGACAACTACTCCCAGCCCCAT -3'
NM_022112	P53AIP1	Forward: 5'- TCCCAGTTTGGTTTCCATGG-3'<br>Reverse: 5'- TCTTCTCTGGCAGGTTTTCCC-3'
NM_001104631	PDE4D	Forward: 5'- GAGGGAACGTGGCATGGA-3'<br>Reverse: 5'- TGAAGCCCACCTGTGATTTTT-3'
AF_029082	Stratifin (14-3-3σ)	Forward: 5'- TCTTCCACTACGAGATCGCCA-3'<br>Reverse: 5'- TTTGTAGGAGTCCTCGCTGAGG-3'
NM_001006946	Syndecan-1	Forward: 5'- GACTTTTCTGCAAACACCAACATG-3'<br>Reverse: 5'- GGAAAGACGAAGGCACAGAGA-3'

NM_003257	TJP1	Forward: 5’- TGCCCAGTGGTATCCAATTGT-3’<br>Reverse:5’-TGGACATAACCTCATTCTCATTGTTT-3’
NM_001071	TYMS	Forward: 5’- GGCCTCGGTGTGCCTTT-3’<br>Reverse: 5’- GATGTGCGCAATCATGTACGT-3’
NM_003380	Vimentin	Forward: 5’- GGCTCGTCACCTTCGTGAAT-3’<br>Reverse: 5’- TCAATGTCAAGGGCCATCTTAA-3’

Supplementary Figure 1. Densitometric analysis data of H4 and tubulin acetylation after ST7612AA1 treament.

Acetyl-H4

Name	Volume	Bg. Value	Bg. Type	Average	Std. Dev.	Sum	Median	Area	SumAbove	PixelsAboveBG
Vehicle	54890,16	856,75	LocalAvg	881,954	136,11	1489090,36	85,4	1674	82650,03	828
SAHA	1160980,7	880,4	LocalAvg	1380,083	534,75	3203520,05	120,09	2320	288385	1930
ST7612 1 µM	1283650,3	863,92	LocalAvg	1372,33	527,55	3460720,24	116,49	2520	338322,2	2415
ST7612 0,2 µM	1331400,5	985,53	LocalAvg	1510,387	605,01	3814940,89	127,46	2520	343753,8	2102
ST7612 0,04 µM	692310,14	995,55	LocalAvg	1384,932	536,72	3501090,87	114,71	2520	307236,9	2060
ST7612 0,008 µM	81970,48	965,98	LocalAvg	1295,126	493,5	3253970,56	108,59	2520	272868,9	1934

H4

Name	Volume	Bg. Value	Bg. Type	Average	Std. Dev.	Sum	Median	Area	SumAbove	PixelsAboveBG
Vehicle	2003504,4	2503,3	LocalAvg	3430,848	1710,46	7410632,35	2638,42	2160	5892486	1527
SAHA	7552463,5	2643,26	LocalAvg	6139,768	5458,79	13261899,63	3173,19	2160	12058226	1681
ST7612 1 µM	3416237,9	2646,8	LocalAvg	4372,17	2665,23	8656897,02	3050,85	1980	7360711	1468
ST7612 0,2 µM	3971440,8	2601,66	LocalAvg	4607,441	3396,27	9122732,54	2971,38	1980	8012467	1534
ST7612 0,04 µM	2239271,2	2593,97	LocalAvg	3522,895	1516,9	6975332,47	2830,97	1980	5750692	1491
ST7612 0,008 µM	3650165,8	2528,32	LocalAvg	4371,837	3160,81	8656238,02	2831,71	1980	7959373	1697

Sample		H4	acetyl H4	Acetyl H4/H4	tubulin	Acetyl tubulin	Acetyl tub./tubulin
Vehicle		2003504	54890,16	0,0274	3643217	1240755,64	0,341
SAHA 5µM		7552464	1160981	0,1537	2217975	5803371,17	2,617
ST7612AA1	1 µM	3416238	1283650	0,3757	4385264	8821349,82	2,012
	0,2 µM	3971441	1331400	0,3352	4047114	4778015,25	1,181
	0,04 µM	2239271	692310,1	0,3092	4696256	3048384,32	0,649
	0,008 µM	3650166	819700,5	0,2246	3379245	1986066,42	0,588

Acetyl-tubulin

Name	Volume	Bg. Value	Bg. Type	Average	Std. Dev.	Sum	Median	Area	SumAbove	PixelsAboveBG
Vehicle	1240755,6	947411	LocalAvg	1593638	1117319	3059784	1049,31	1920	2478948.1[illegible]	1240
SAHA	5803371,2	1293072	LocalAvg	4590442	4403790	8079177,99	2426,75	1760	7509078.4[illegible]	1197
ST7612 1 µM	8821349,8	1051420	LocalAvg	4939438	2927469	8949423,08	2510,8	2024	8372993.8[illegible]	1397
ST7612 0,2 µM	4778015,3	938010	LocalAvg	2630782	2884803	6182338,5	1155,06	2350	5530688.1[illegible]	1566
ST7612 0,04 µM	3048384,3	910238	LocalAvg	2290222	2388619	5059100,49	1150,85	2209	4542391.8[illegible]	1571
ST7612 0,008 µM	1986066,4	846306	LocalAvg	1745385	1693459	3855556,52	978,18	2209	3486563.2[illegible]	1732

tubulin

Name	Volume	Bg. Value	Bg. Type	Average	Std. Dev.	Sum	Median	Area	SumAbove	PixelsAboveBG
Vehicle	3643216,6	808403	LocalAvg	2537504	3295948	5346520,88	955.02	2107	5023132.6[illegible]	1669
SAHA	2217975,2	947147	LocalAvg	3282682	2990373	5928523,06	1239.04	1806	5576049.5[illegible]	1379
ST7612 1 µM	4385263,9	901774	LocalAvg	3067336	3840894	6211356,1	1132.67	2025	5939926.2[illegible]	1692
ST7612 0,2 µM	4047113,5	894150	LocalAvg	2892724	3577212	5857766,73	1119.25	2025	5569611.6[illegible]	1671
ST7612 0,04 µM	4696256,4	864943	LocalAvg	2690255	3382673	5447766,53	1104.99	2025	5187762.1[illegible]	1692
ST7612 0,008 µM	3379244,8	828106	LocalAvg	2728820	3429577	5287200,54	1013.00	2304	4996128.6[illegible]	1924

Supplementary Figure 2. Antiproliferative activity of ST7612AA1 on some tumor cell lines at different times.

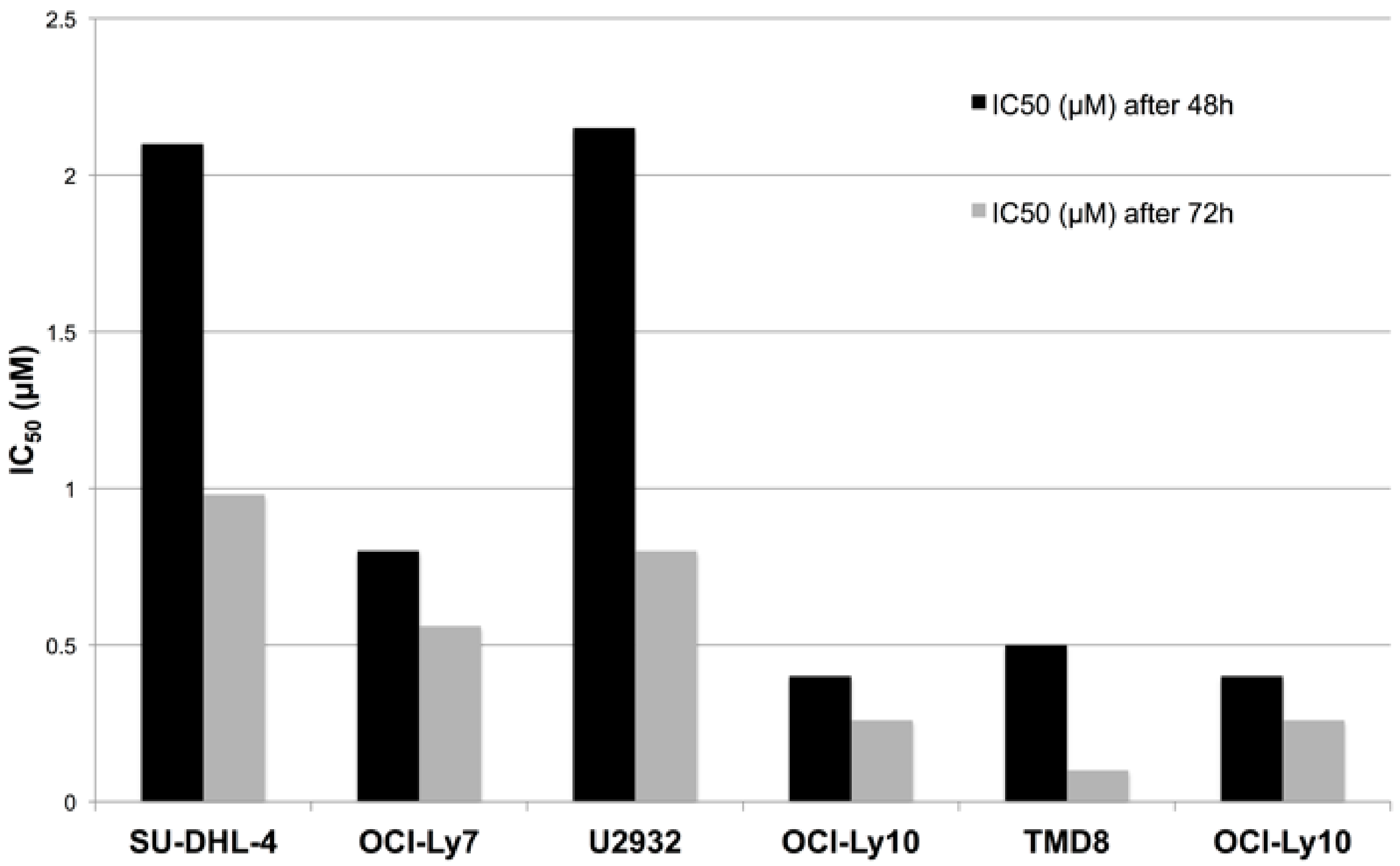

Supplementary Figure 3. Validation of GEP results by real-time qPCR analysis.

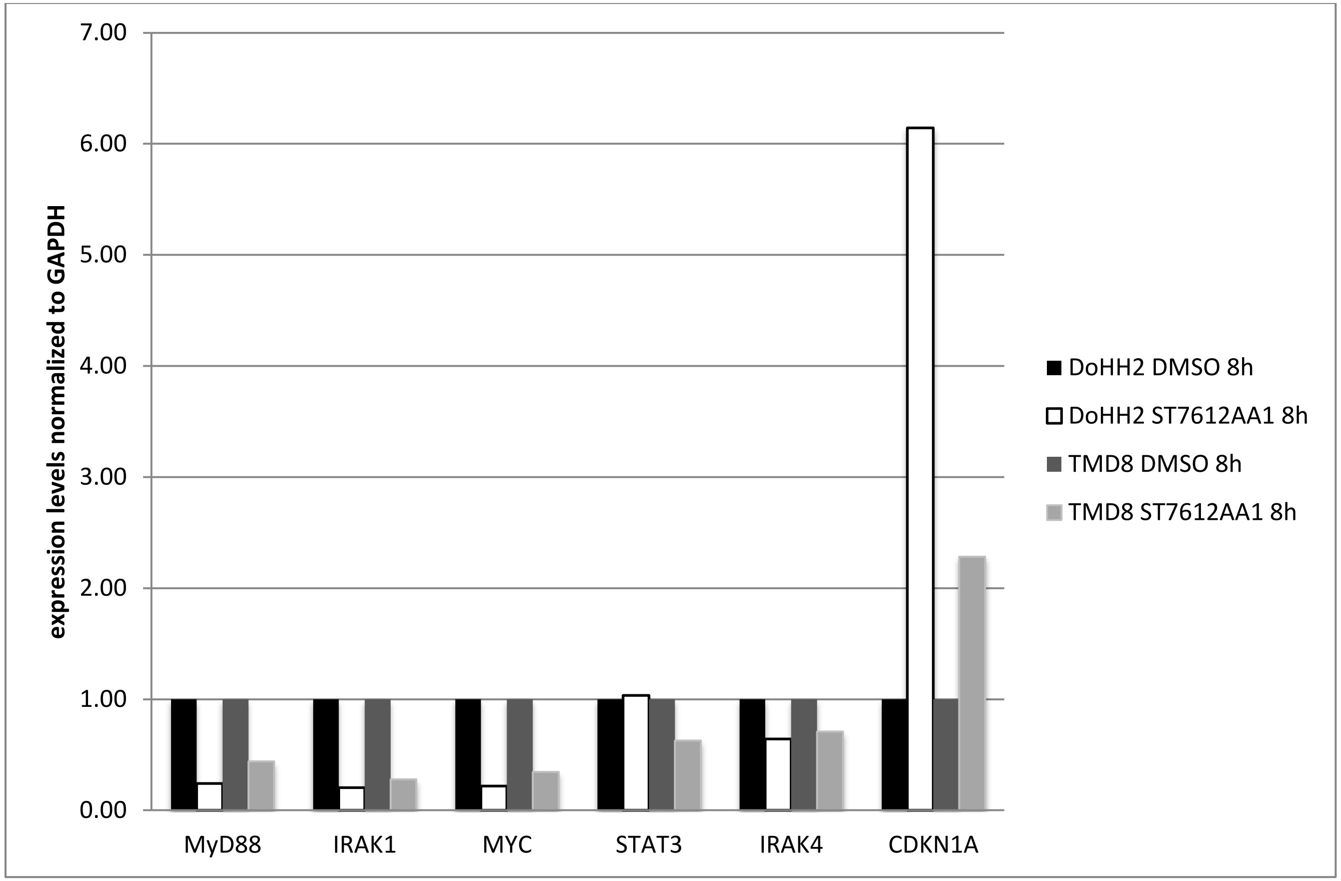

Supplementary Figure 4. Antitumor effect of ST7612AA1 against DOHH2 lymphoma xenografted in nude mice.

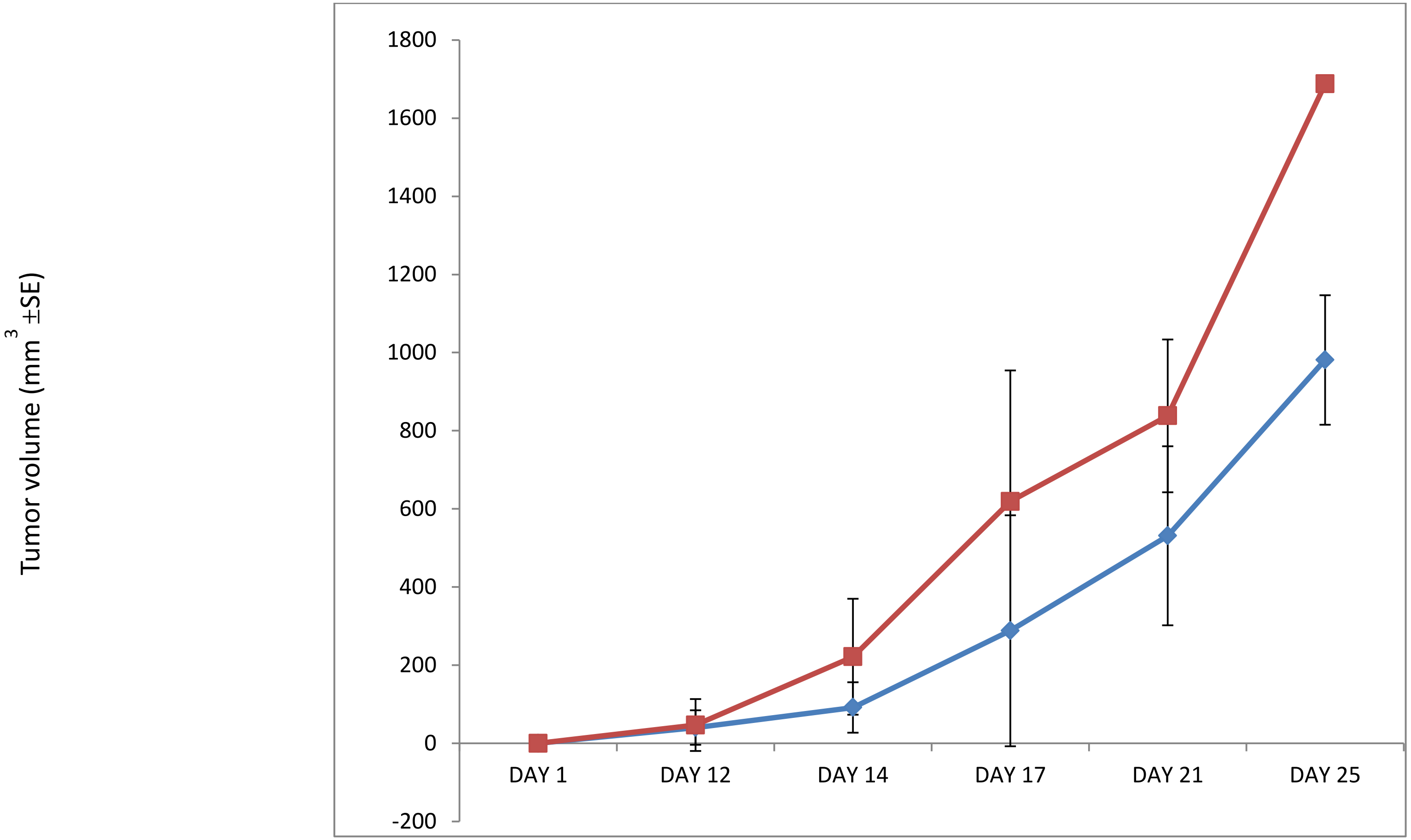

Supplement: Supplementary file 1 [file oncotarget-06-5735-s001.pdf]
